# A tailored mouse model of CLN2 disease: A nonsense mutant for testing personalized therapies

**DOI:** 10.1371/journal.pone.0176526

**Published:** 2017-05-02

**Authors:** Ryan D. Geraets, Logan M. Langin, Jacob T. Cain, Camille M. Parker, Rosanna Beraldi, Attila D. Kovacs, Jill M. Weimer, David A. Pearce

**Affiliations:** 1 Children’s Health Research Center, Sanford Research, Sioux Falls, South Dakota, United States of America; 2 Sanford School of Medicine at the University of South Dakota, Sioux Falls, South Dakota, United States of America; University of Cologne, GERMANY

## Abstract

The Neuronal Ceroid Lipofuscinoses (NCLs), also known as Batten disease, result from mutations in over a dozen genes. Although, adults are susceptible, the NCLs are frequently classified as pediatric neurodegenerative diseases due to their greater pediatric prevalence. Initial clinical presentation usually consists of either seizures or retinopathy but develops to encompass both in conjunction with declining motor and cognitive function. The NCLs result in premature death due to the absence of curative therapies. Nevertheless, preclinical and clinical trials exist for various therapies. However, the genotypes of NCL animal models determine which therapeutic approaches can be assessed. Mutations of the *CLN2* gene encoding a soluble lysosomal enzyme, tripeptidyl peptidase 1 (TPP1), cause late infantile NCL/CLN2 disease. The genotype of the original mouse model of CLN2 disease, *Cln2*^*-/-*^, excludes mutation guided therapies like antisense oligonucleotides and nonsense suppression. Therefore, the purpose of this study was to develop a model of CLN2 disease that allows for the assessment of all therapeutic approaches. Nonsense mutations in CLN2 disease are frequent, the most common being *CLN2*^*R208X*^. Thus, we created a mouse model that carries a mutation equivalent to the human p.R208X mutation. Molecular assessment of *Cln2*^*R207X/R207X*^ tissues determined significant reduction in *Cln2* transcript abundance and TPP1 enzyme activity. This reduction leads to the development of neurological impairment (e.g. tremors) and neuropathology (e.g. astrocytosis). Collectively, these assessments indicate that the *Cln2*^*R207X/R207X*^ mouse is a valid CLN2 disease model which can be used for the preclinical evaluation of all therapeutic approaches including mutation guided therapies.

## Introduction

Children across the globe are affected by any one of more than 40 different lysosomal storage disorders (LSDs); many of them resulting from deficiencies in either a lysosomal membrane protein or lysosomal enzyme [1, 2]. These deficiencies affect normal lysosomal function resulting in an accumulation of various lysosomal substrates. LSDs are commonly categorized according to the constituents of this accumulated material thus resulting in generalized LSD categories; for example the mucopolysaccharidoses accumulate mucopolysaccharides [1, 2]. All LSDs affect multiple physiological systems however, most appear to illicit a significant effect on the central nervous system (CNS) [3–5].

The Neuronal Ceroid Lipofuscinoses (NCLs) are a special category of LSD and accumulate a storage material referred to as ceroid lipofuscin [6–8]. Most commonly, NCL onset begins in childhood however in rarer occasions onset can occur in adulthood. Symptomatic hallmarks of the NCLs consist of but are not limited to epilepsy, tremors, retinopathy, gross and fine motor deterioration, and cognitive decline. Given the large neurological manifestations and predominance in childhood onset, the NCLs are considered to be the most common pediatric neurodegenerative disease [9–12]. Unfortunately, the NCLs are not limited to a single gene or genetic mutation. Currently, several mutations have been identified in more than 13 different genes; all resulting in a specific form of NCL [12–15]. Generally, these NCL forms are based on disease onset and mutated gene. The most common altered genes are *CLN1*, *CLN2*, and *CLN3* causing classic Infantile Neuronal Ceroid Lipofuscinosis (cINCL), classic Late Infantile Neuronal Ceroid Lipofuscinosis (cLINCL), and classic Juvenile Neuronal Ceroid Lipofuscinosis (cJNCL) respectively [9, 16, 17].

cLINCL, also referred to as CLN2 disease, stems from a deficiency in the lysosomal enzyme tripeptidyl peptidase 1 (TPP1). This deficiency leads to a disease onset ranging from 2 to 4 years of age with initial clinical symptoms consisting of developmental delay and epilepsy [9, 16, 18]. Like all other NCLs, cLINCL results in premature death. To combat this premature death, numerous therapeutic approaches have been hypothesized and assessed [19, 20]. Experimental and clinical trials for cLINCL focus on gene therapy and enzyme replacement therapy. However, the assessment of some therapeutic approaches (i.e. antisense oligonucleotides and nonsense suppression compounds) is limited by the genotypes of available cLINCL animal models; for example, the genotype of the commonly used *Cln2*^*-/-*^ mouse model does not permit the assessment of significantly relevant mutation-targeted therapies. Therefore, the purpose of this study was to generate an alternative cLINCL animal model that would allow for the assessment of all therapeutic approaches including nonsense suppression therapies. The most commonly annotated *CLN2* mutations are c.509-G>C and p.R208X [21, 22]. Since one of the two most common mutations is a nonsense mutation, we generated a mouse model that carries the equivalent p.R208X mutation. Amino acid sequence for murine TPP1 is shorter than human TPP1 by 1 amino acid which occurs prior to the mutation; thus, the new cLINCL animal model is referred to as *Cln2*^*R207X/R207X*^. To be considered a valid model of cLINCL, *Cln2*^*R207X/R207X*^ mice were vigorously assessed using various molecular, histological, and behavioral assessments. Results indicate that the *Cln2*^*R207X*^ mutation leads to a significant reduction in both *Cln2* transcript level and TPP1 enzyme activity. Consequences of these reductions consist of hallmark histopathological changes (i.e. substrate accumulation and astrocytosis) and neurological deficits (i.e. declining motor skills and tremors).

## Materials and methods

### Ethics statement

All animal research carried out in this manuscript followed National Institute of Health (NIH) and Sanford Research Institutional Animal Care and Use Committee (IACUC) guidelines. The animal protocol for this study was reviewed and approved by the Sanford Research IACUC.

### Animals

Mice heterozygous for the *Cln2* c.619C>T mutation in exon 6 (Cln2 p.R207X) and the *neo* cassette, *Cln2*^*R207X;neo/+*^, were generated and obtained from Applied Stem Cell, Inc. Generation of the *Cln2*^*R207X;neo/+*^mice was similar to the previously reported *Cln1*^*R151X;neo/+*^ mice [23]. Conversely, the *neo* cassette in the *Cln2*^*R207X;neo/+*^mice were flanked with FRT-sites. *Cln2*^*R207X;neo/+*^mice were natural bred to produce *Cln2*^*R207X;neo/R207X;neo*^. *Cln2*^*R207X;neo/R207X;neo*^ mice were crossed with FLPo-10 mice (The Jackson Laboratory) to produce *Cln2*^*R207X/+*^ mice. Flippase-mediated excision of the *neo* cassette was assessed using polymerase chain reaction (Forward primer: 5’-GCTATTCGGCTATGACTGGG–3’; Reverse primer: 5’–CCCGGTAGAATTCCGATCAT-3’). Heterozygous *Cln2*^*R207X/+*^ mice were bred to produce colonies of *Cln2*^*R207X/R207X*^ and *Cln2*^*+/+*^. Confirmation genotypic screening was performed using a quantitative real-time polymerase chain reaction assay (Forward primer: 5’- TGCCTGAGCCCACATCTTTG-3’; Reverse primer: 5’-TGGGAGTGACTCCGTCTGT-3’; Reporter 1(VIC): 5’- CAGGTTGTATCGCTGACG-3’; Reporter 2(FAM): 5’- CAGGTTGTATCACTGAC-3’) from Thermo Fisher Scientific. Running conditions for the qRT-PCR assay consisted of: 1) 95°C for 15 min. (1 cycle) and 2) 95°C for 1 sec. and 60°C for 1 min. (40 cycles). Genomic sequencing confirmation was conducted through Eurofins Genomics (Forward primer: 5’-CTTGCTGGCTGAGTGTCCGGT-3’; Reverse primer: 5’-CTACCAGCTTCCCCAGGCCTTG-3’). Both the *Cln2*^*R207X/R207X*^ and *Cln2*^*+/+*^ colonies were on a mixed genetic background (C57BL/6x129S6/SvEv). All animals were housed, cared for, and used under the guidelines set by the NIH and the direction of the Sanford Research IACUC. Mice were housed in individually vented microisolator cages (four or five mice/cage) with ad libitum access to food and water. Mice were fed with the Teklad Global 2918 diet (Harlan Laboratories, Indianapolis, IN), and their drinking water was tap water.

*Cln2*^*R207X/R207X*^ mice have a severe disease phenotype, twitching starts at 90 days of age and die within a couple months after twitching onset. Therefore, from 90 days of age, *Cln2*^*R207X/R207X*^ mice were monitored daily and were euthanized by carbon dioxide when debilitating phenotypes (i.e. immobility, difficulties in feeding) were observed. The care for the *Cln2*^*R207X/R207X*^ mice followed our animal protocol approved by the Sanford Research IACUC.

### Protein sample preparation

Under the NIH and Sanford Research IACUC guidelines, 1-month-old *Cln2*^*R207X/R207X*^ (n = 3) and *Cln2*^*+/+*^ (n = 3) mice were euthanized using a carbon dioxide chamber. Following euthanasia, animals were flushed with 10 ml of sterile phosphate buffered saline solution via cardiac perfusion. Tissues including cerebral cortex, cerebellum, liver, spleen, and kidney were harvested and flash frozen using dry ice. Tissues were stored at -80°C. Upon their removal from -80°C, tissue sections were removed using a sterile blade, placed into 2mL tubes containing zirconium beads and 500 μL lysis buffer [50 mM Tris-HCl pH 7.4; 150 mM NaCl; 0.2% Triton X-100; 0.3% NP-40; 0.1 mM PMSF; 1x HALT Protease Inhibitor Cocktail (Thermo Fisher Scientific)], and then homogenized using a Bertin Technologies Precellys 24 homogenizer. Samples were then transferred to clean 1.5-ml microcentrifuge tubes and pulse sonicated using a Branson Sonifier 250. Following sonication, samples were incubated on ice for 30 min and then centrifuged at 12,000g for 10min. Sample supernatants were then transferred to clean pre-chilled 1.5mL microcentrifuge tubes and stored at -80°C. Total protein concentrations were quantified using the Pierce 660 nm Protein Assay (Thermo Fisher Scientific).

### TPP1 enzyme activity assay

Using previously described methods, the aforementioned *Cln2*^*R207X/R207X*^ and *Cln2*^*+/+*^ total protein samples were evaluated for TPP1 enzyme activity [24, 25]. The assays were conducted in a black, flat-bottom 96-well plate. 10 μg of total protein were loaded into each well. Each sample was done in quadruplicates. 40 μl of 250 μM Ala-Ala-Phe-7-amido-4-methylcoumarin [10 mM Ala-Ala-Phe AMC (Sigma Aldrich) diluted in substrate buffer (150 mM NaCl; 0.1% Triton X-100; 100 mM sodium acetate; pH 4.0)] was added to each well. The plate was incubated in the dark at 37°C for 1h. 200 μl of stop buffer [0.1 M monochloroacetic acid; 0.13 M NaOH; 0.1 M acetic acid; pH 4.3] was added to each well. The plate was gently shaken and fluorescence was read using a BioTek Cytation 3 (Excite 380 nm/Emit 460 nm). Background fluorescence was accounted and removed from each sample. Relative fluorescence was calculated as a percentage of wild type fluorescence.

### PPT1 enzyme activity assay

Using previously described methods, the aforementioned *Cln2*^*R207X/R207X*^ and *Cln2*^*+/+*^ total protein samples were evaluated for PPT1 enzyme activity [25, 26]. The assays were conducted in a black, flat-bottom 96-well plate. 10 μg of total protein were loaded into each well. Each sample was done in quadruplicates. 20 μl of PPT1 substrate [0.64 mM 4-methylumbelliferyl-6-thiopalmitoyl-β-glucoside (Santa Cruz); 15 mM dithiothreitol with 2.3 mg/ml bovine serum albumin and 0.09% sodium azide; 0.2 M disodium phosphate with 0.1 M citric acid buffer pH 4.0; 0.945 mg/mL β-Glucosidase from almonds (Sigma Aldrich)] was added to each well. The plate was incubated in the dark at 37°C for 1h. 200 μl of stop buffer [0.5 M sodium bicarbonate with 0.5 M sodium carbonate pH 10.7; 0.025% Triton X-100] was added to each well. The plate was gently shaken and fluorescence was read using a BioTek Cytation 3 (Excite 355 nm/Emit 460 nm). Background fluorescence was accounted and removed from each sample. Relative fluorescence was calculated as a percentage of wild type fluorescence.

### Sphingomyelinase activity assay

Sphingomyelinase activity was assessed using the Amplex Red Sphingomyelinase Assay Kit (Thermo Fisher Scientific). The assay was performed according to the kits protocol. 10 μg of total protein was assessed for each sample and done in triplicates. Fluorescence was read using a BioTek Cytation 3 (Excite 545 nm/Emit 590 nm). Background fluorescence was accounted and removed from each sample. Relative fluorescence was calculated as a percentage of wild type fluorescence.

### RNA sample preparation

Under the NIH and Sanford Research IACUC guidelines, 1-month-old *Cln2*^*R207X/R207X*^ (n = 3) and *Cln2*^*+/+*^ (n = 3) mice were euthanized using a carbon dioxide chamber. Following euthanasia, animals were flushed with 10 ml of sterile phosphate buffered saline solution via cardiac perfusion. Tissues including cerebral cortex, cerebellum, liver, spleen, and kidney were harvested and flash frozen using dry ice. Tissues were stored at -80°C. Upon their removal from -80°C, tissue sections were removed using a sterile blade and placed into RNAse free tubes. Maxwell 16 LEV simplyRNA tissue kits and a Maxwell 16 instrument (Promega) were utilized for RNA extractions. Samples were processed according to the Maxwell 16 LEV simplyRNA tissue kit. RNA samples were then assessed and quantified using the BioTek Epoch Microplate Spectrophotometer; all samples had concentrations between 200–1000 ng/μl and an A_260_/A_280_ > 2.

### Reverse transcription

Using the GoScript Reverse Transcription System (Promega), cDNA synthesis was performed on 1 μg of total RNA from each sample. Reactions were run in an Applied Biosystems Veriti Thermal Cycler with the following conditions: 25°C for 5 min., 42°C for 60 min., 70°C for 15 min., and a 4°C hold. Samples were stored at -20°C.

### Quantitative real-time polymerase chain reaction

Prior to their use, cDNA samples were diluted 1:20 using nuclease free water. Quantitative real-time polymerase chain reactions were carried out using the Absolute Blue QPCR Mix (Thermo Fisher Scientific). *Cln2* expression was measured using the *Tpp1* TaqMan Gene Expression Assay from Thermo Fisher Scientific (Mm00487016_m1). In addition, *Gapdh* expression was measured using the *Gapdh* TaqMan Gene Expression Assay from Thermo Fisher Scientific (Mm99999915_g1). All reactions were run in a Stratagene Mx3005P qPCR machine (Agilent Technologies). Reaction conditions consisted of the following: 1) 95°C for 15 min. (1 cycle) and 2) 95°C for 15 sec.; 60°C for 1 min. (40 cycles). ΔΔCT was used to calculate relative fold expression.

### Behavioral testing

All behavioral tests were performed on male mice as they became available in our animal colony. The same cohorts of *Cln2*^*R207X/R207X*^ (n = 20) and *Cln2*^*+/+*^ (n = 14) mice were tested at 1 and 3 months of age. Prior to performing any behavioral tests, the mice were acclimated to the environment for at least 20 min. Behavioral testing consisted of the following four day scenario: Day 1) Modified vertical pole test and force-plate actimeter, Day 2) Rotarod, Day 3) Light/Dark box, and Day 4) Hanging wire.

### Modified vertical pole test

Vertical pole testing using a threaded metal rod was carried out in two phases: 1) Climbing downward and 2) Turning downward; according to our published method [23, 27]. First, the mouse was placed at the top of the pole facing downward, and was given a 60-s window to descend the pole. The time to descend the pole was recorded and tracked for 5 consecutive trials. After the 5^th^ climbing down trial, the same mouse was placed towards the top of the pole facing upwards and timed to determine how long it takes to turn around and face downward. The mouse was given 4 consecutive trials and a 60-s window per trial. If the mouse fell from the pole during the climbing down or turning downward trials the mouse was given a time of 60 sec.

### Force-plate actimeter

Mice were placed into a force-plate actimeter (BASi, West Lafayette, IN) and allowed to roam for 20 min. All tracked information was processed and analyzed using FPAanalysis software version 1.10.01 (BASi, West Lafayette, IN).

### Rotarod test

An accelerating rotarod test was administered as we described previously [23, 27]. Rota Rod Rotamex-5 machines (Columbus Instruments, Columbus, OH) were used for animal testing. Testing parameters consisted of the following: 1) start speed: 0 rpm; end speed: 48 rpm, 2) acceleration occurred in increments of 0.2 rpm per second, and 3) total allotted test time: 240 s. Mice were trained on the rotarod in 3 consecutive trial runs. After a 1.5-h resting period, mice were tested on the rotarod in 3 test trials each consisting of three consecutive runs, with 15 minutes of rest between trials. Latency to fall was calculated as an average of the 9 test runs.

### Light/Dark box test

Anxiety was measured in 3-month-old *Cln2*^*R207X/R207X*^ and *Cln2*^*+/+*^ mice via the light/dark box test [28, 29]. Mice were placed into light/dark boxes (Stoelting Co., Wood Dale, IL) and allowed to roam freely for 20 min. Mouse movement was tracked and recorded using ANY-maze equipment and software (Stoelting Co., Wood Dale, IL). The recorded data was used to determine the amount of time spent in the dark and the amount of time spent in the light.

### Hanging wire test

Strength in addition to motor coordination was assessed using a modified hanging wire test [29, 30]. Only 3-month-old *Cln2*^*R207X/R207X*^ and *Cln2*^*+/+*^ mice were tested. To conduct this test, mice were placed in the center of a food rack which was subsequently inverted and suspended approximately 18 inches above a cage containing bedding. Mice were timed to see how long they could remain suspended; 60 s was used as the cut-off time. Each mouse was tested 5 times and the average time suspended was calculated.

### Histological preparation

Under the NIH and Sanford Research IACUC guidelines, 3-month-old *Cln2*^*R207X/R207X*^ and *Cln2*^*+/+*^ mice were euthanized using a carbon dioxide chamber. Following euthanasia, animals were flushed with 10 ml of sterile phosphate buffered saline solution via cardiac perfusion. PBS perfusion was followed by a 10 ml perfusion of 4% paraformaldehyde in PBS. Tissues including the cerebrum and cerebellum were harvested and placed into individual scintillation vials containing a solution of 4% paraformaldehyde in PBS. After 24 h, the 4% paraformaldehyde solution was replaced with PBS containing 0.02% sodium azide and stored at 4°C. Tissues were removed from storage and embedded into 3% low melting point agarose. Samples were sliced into 50μm sections using a Leica VT1000S vibratome and serially divided 1:6.

### Immunohistochemistry

Serial sections of *Cln2*^*R207X/R207X*^ (n = 5) and *Cln2*^*+/+*^ (n = 4) cerebrums were immunostained for mitochondrial ATP synthase subunit c (subunit c), glial fibrillary acidic protein (GFAP), and ionized calcium binding adapter molecule 1 (Iba1). Serial sections of *Cln2*^*R207X/R207X*^ (n = 5) and *Cln2*^*+/+*^ (n = 4) cerebella were immunostained for mitochondrial ATP synthase subunit c (subunit c). All immunostainings were done on floating tissue sections and conducted in a 24-well plate. Incubations lasting an hour or more were done on a plate rocker. Tissue sections were blocked in blocking buffer [PBS; 3% bovine serum albumin; 5% goat serum; 0.2% Triton X-100; 0.02% sodium azide] for 1 h at room temperature. Blocking buffer was removed and sections were incubated with the primary antibody diluted in blocking buffer [anti-subunit c 1:100 (Abcam: ab181243); anti-GFAP 1:250 (Dako: 70334); anti-Iba1 1:250 (Biocare Medical: CP290 A,B)] at room temperature overnight. Primary antibody was removed and slices were washed three times in PBS. The secondary antibody [anti-rabbit IgG coupled to Alexa Fluor 568 (Thermo Fisher Scientific: z25306)] diluted 1:1500 and 4’,6-Diamidino-2-Phenylindole (DAPI) (Thermo Fisher Scientific: D1306) diluted 1:1000 all in PBS was added to each sample. The plates were covered in aluminum foil and incubated at room temperature for 1 h. The secondary antibody and DAPI were removed and slices were washed three times in PBS. Floating sections were then transferred to labeled glass slides, mounted using DAKO mounting medium, and covered with cover glass. Slides were allowed to dry at room temperature and then stored at 4°C.

Double staining for co-localization was conducted using the same protocol. Anti-mitochondrial ATP synthase subunit c 1:100 (Abcam: ab181243) and anti-lysosomal associated membrane protein 1 (LAMP-1) 1:1000 (Santa Cruz: sc-20011) diluted in blocking buffer were used as the primary antibodies. The secondary antibodies were: anti-rabbit IgG coupled to Alexa Flour 568 (Thermo Fisher Scientific: z25306) 1:1500 and anti-mouse IgG coupled to Alexa Fluor 488 (Thermo Fisher Scientific: z25002) 1:1500.

### Image acquisition

All slides were viewed and images collected using a Nikon Eclipse Ni-E microscope equipped with Photometrics CoolSNAP MYO CCD camera. Cerebral and cerebellar sections were viewed and imaged using a 20X-objective. Cortical regions of cerebral sections were visually divided into superficial and deep cortical layers. Four images were collected per superficial and deep cortical layers per cerebral section. This approach was used for all cerebral immunostainings. Sections double immunostained for co-localization were viewed and imaged using an oil immersion 100X objective.

### Image analysis

Images were analyzed and processed with the Cellomics Scan (Thermo Fisher Scientific) software using the Spot Detector BioAssay (Version 4). Subunit c positive staining was measured in a region of interest created based on the DAPI signal and expanded by 10 pixels using a threshold based analysis. Individual cells were then identified and counted based on the nuclei (DAPI), subunit c signal intensity was then measured in the region of interest and positive puncta were counted and measured for size. Averages of 6000 cells were counted per group. In GFAP analysis, cells stained positive for GFAP were used to measure the total staining intensity, the total area of GFAP staining, and the average area of a GFAP positive cell. In Iba1 analysis, cells stained positive for Iba1 were used to measure the total staining intensity, the average area of a Iba1 positive cell, and the average staining intensity per cell.

### Statistical analysis

All data was graphed and statistically analyzed using GraphPad PRISM Version 5.04 software.

## Results

### *CLN2* mutations

**Fig 1:**
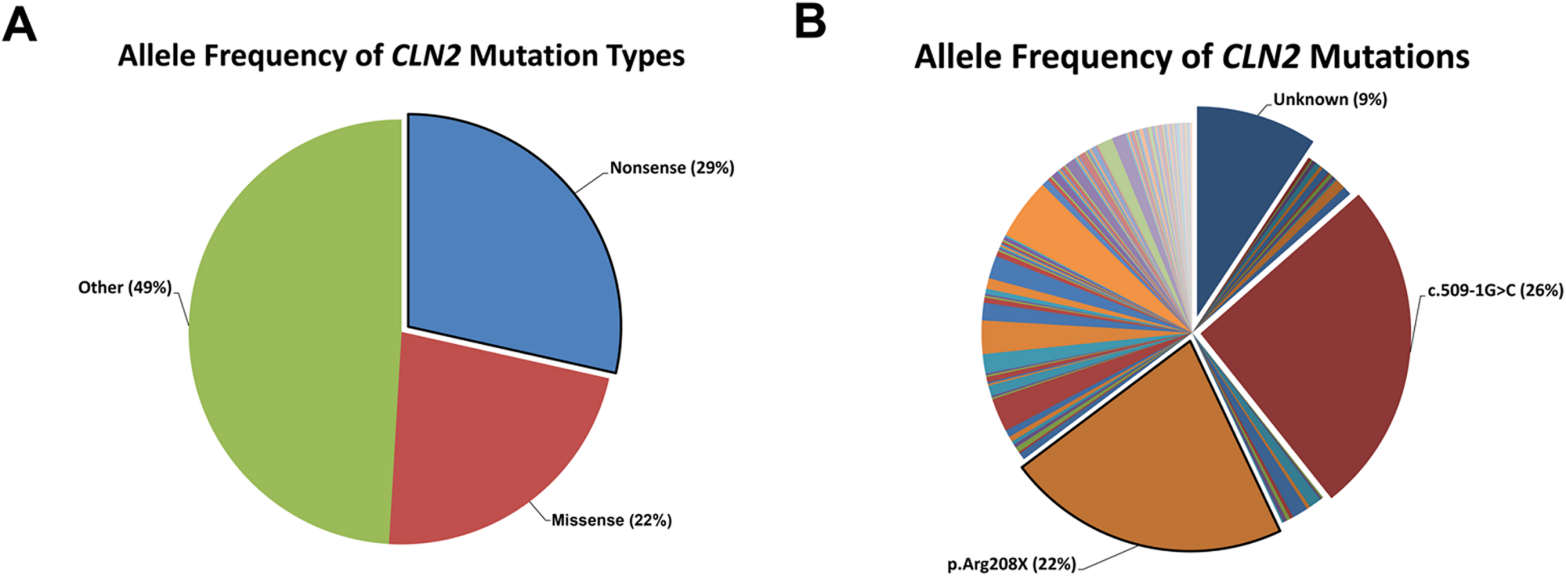
Frequency of CLN2 disease-associated mutations. (A) Allele frequency of CLN2 disease mutations demonstrates a predominance of nonsense (29%) and missense (22%) mutations. (B) The most common CLN2 disease mutations consists of either the intronic transversion c.509-1G>C that results in altered transcript splicing or the exonic transition c.622C>T that results in the p.R208X nonsense mutation.

To establish mutation type frequencies (e.g. missense, nonsense) in CLN2 disease patients, the University College London’s online NCL mutation database was assessed for recorded *CLN2* mutations [22]. Missense and nonsense mutations account for more than 50% of the recorded mutation allele frequencies; of which missense mutations contribute 22% and nonsense mutations contribute 29% (Fig 1A). Using the same set of data, we determined that the most common nonsense mutation in CLN2 disease patients is p.Arg208X; which appears to occur with an allele frequency of 22% (Fig 1B). This correlates with previous reports on the occurrence of p.Arg208X [21, 31]. The allele frequency of *CLN2* p.Arg208X directed us to generate the murine equivalent, *Cln2*^*R207X*^.

### *Cln2*^*R207X/R207X*^ mouse model generation

**Fig 2:**
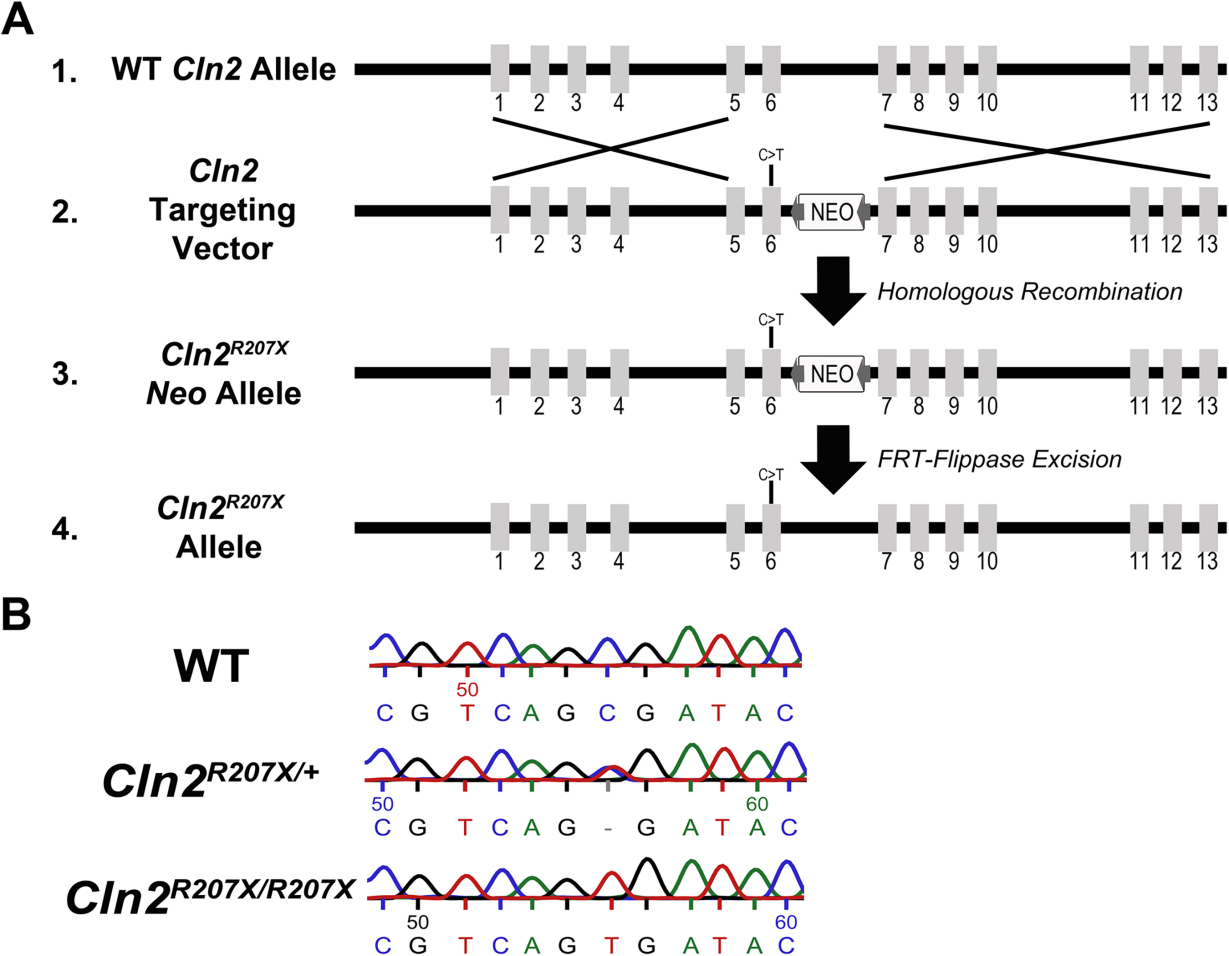
Genetic design of the CLN2 disease mouse model, *Cln2*^*R207X/R207X*^. (A) A *Cln2* targeting vector carrying the c.619C>T mutation (Line 2), also known as p.R207X mutation, was electroporated into C57BL/6x129S6/SvEv embryonic stem cells. Homologous recombination between wildtype *Cln2* (Line 1) and the targeting vector (Line 2) resulted in the generation of a *Cln2*^*Neo;R207X*^ allele (Line 3). Removal of the *Neo* cassette via FRT-flippase mediated excision (FRT sites represented by grey arrows flanking the *Neo* cassette) generated the *Cln2*^*R207X*^ allele (Line 4) resulting in *Cln2*^*+/R207X*^ mice. *Cln2*^*+/R207X*^ mice were bred to create *Cln2*^*R207X/R207X*^ mice. (B) *Cln2* sequencing results confirm the retention of the mutation following breeding.

Complete overview of the *Cln2*^*R207X/R207X*^ mouse model generation is represented in Fig 2A. Applied StemCell, Inc. was outsourced to generate our initial *Cln2*^*R207XNeo/+*^ mouse founders. To create these founders, a *Cln2* targeting vector carrying a C to T transition in exon 6 and a Neomycin selection cassette flanked with FRT sites between exon 6 and exon 7, all within *Cln2*, was placed into mouse embryonic stem cells. Through homologous recombination the targeting vector inserted into the embryonic stem cell genome. *Cln2*^*R207XNeo/+*^ mouse founders were then bred to create *Cln2*^*R207XNeo/R207XNeo*^. The Neomycin selection cassette was removed via FRT-Flippase mediated excision by breeding *Cln2*^*R207XNeo/R207XNeo*^ mice with FLPo-10 mice obtained from Jackson Laboratory. The *Cln2*^*R207X/+*^ mice obtained following Neomycin cassette removal were then used to generate our *Cln2*^*+/+*^ and *Cln2*^*R207X/R207X*^ colonies. Sequencing results confirmed preservation of the point mutation (Fig 2B).

### *Cln2*^*R207X*^ transcript level and TPP1 enzyme activity in *Cln2*^*R207X/R207X*^ mice

**Fig 3:**
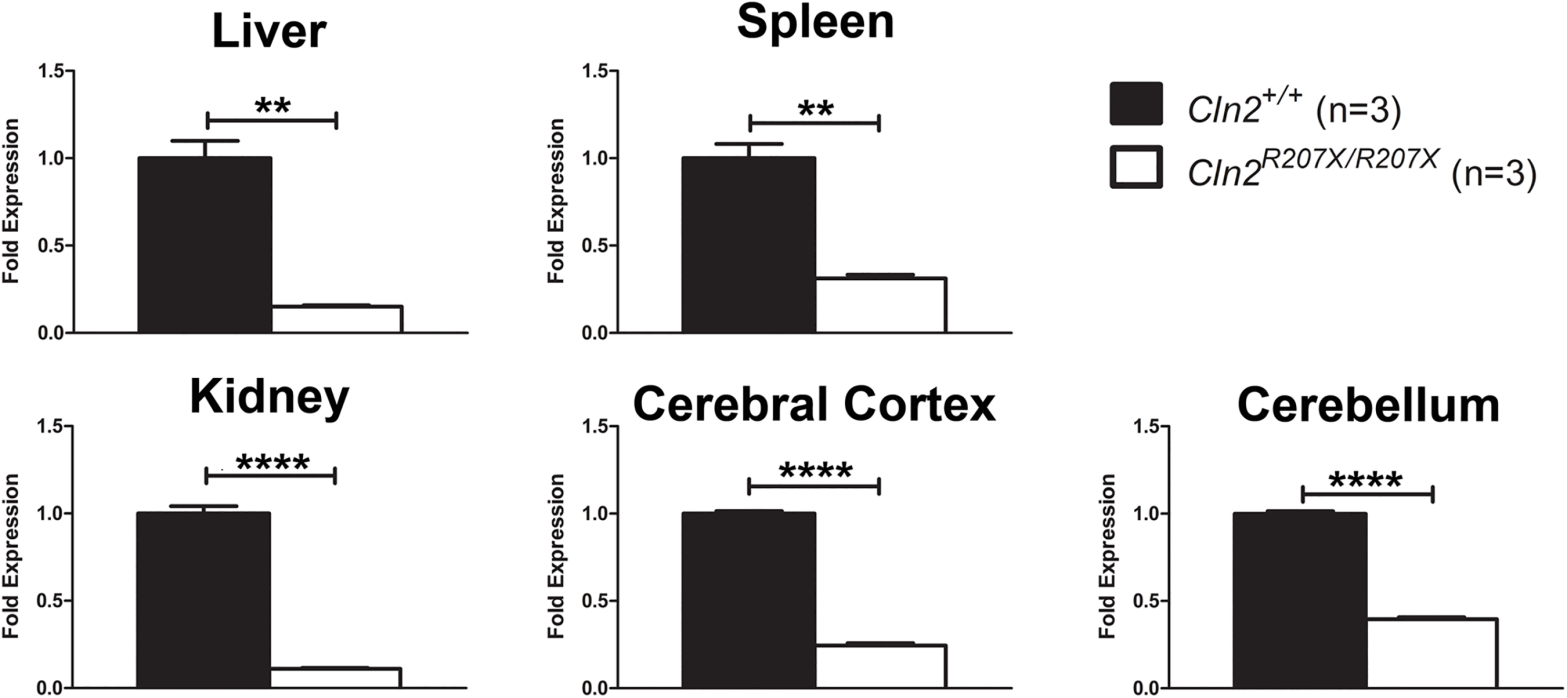
Ubiquitously reduced *Cln2* transcript abundance in *Cln2*^*R207X/R207X*^ mice. Quantitative real-time PCR was used to measure endogenous levels of the *Cln2* transcript from five different tissues obtained from 1-month-old *Cln2*^*+/+*^ (n = 3) and *Cln2*^*R207X/R207X*^ (n = 3) mice. Three technical replicates were performed using the three biological samples obtained from *Cln2*^*+/+*^ and *Cln2*^*R207X/R207X*^ mice. *Cln2* transcript levels were normalized to *Gapdh* expression. Columns and bars represent mean ± SEM. Statistical significance was determined using an unpaired t-test (**p < 0.01 and **** p < 0.0001).

Transcripts containing nonsense mutations/premature termination codons (PTCs) are targeted for degradation via the nonsense mediated decay (NMD) pathway; thus minimalizing the abundance of PTC-containing transcripts and reducing the production of truncated and potentially harmful proteins. To assess NMD in *Cln2*^*R207X/R207X*^ mice, *Cln2* transcript levels were measured from liver, spleen, kidney, cerebral cortex, and cerebellum via quantitative real-time PCR (qRT-PCR). *Cln2*^*R207X/R207X*^ mice demonstrated a 60–90% reduction in *Cln2* transcript levels when compared to *Cln2*^*+/+*^ mice (Fig 3).

**Fig 4:**
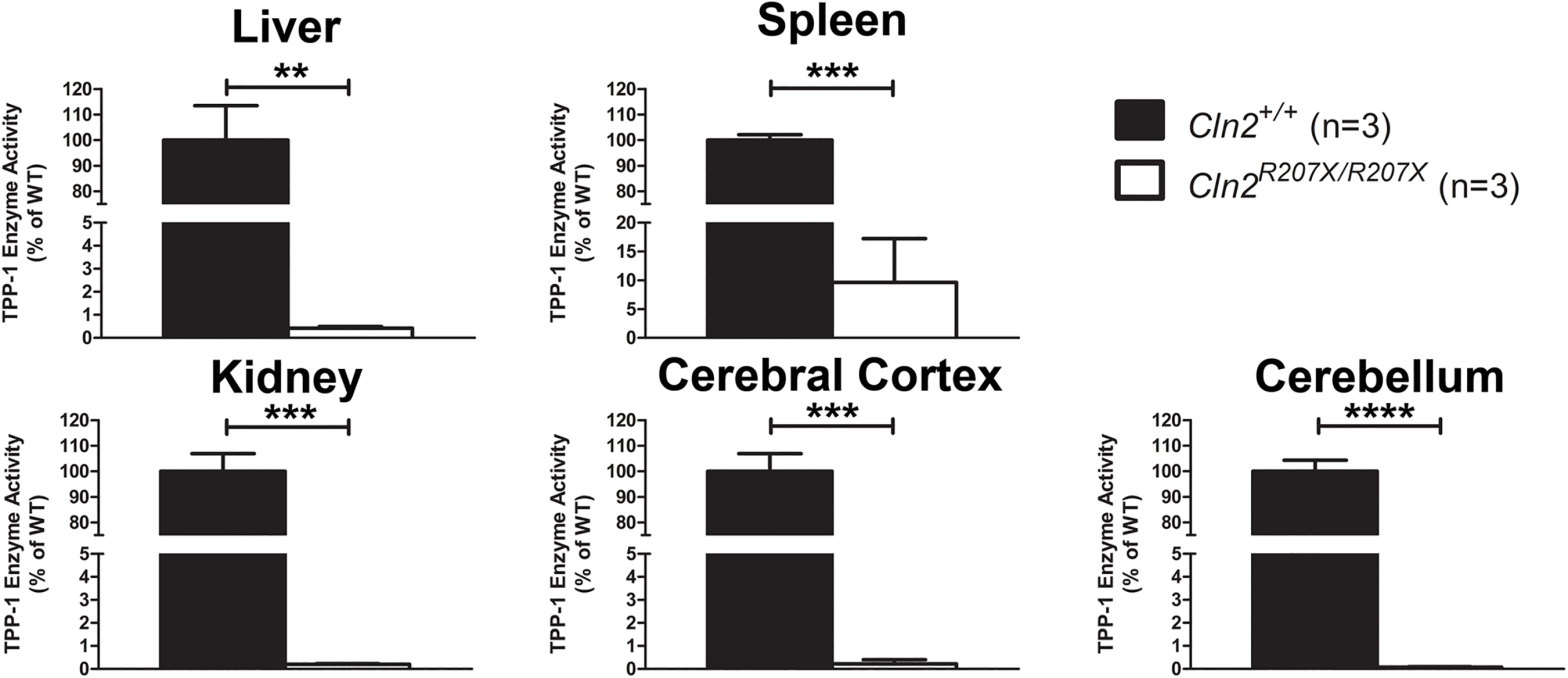
Decreased TPP1 activity in various *Cln2*^*R207X/R207X*^ mouse tissues. Fluorogenic TPP1 enzyme activity assays were used to measure endogenous levels of TPP1 activity from five different tissues obtained from 1-month-old *Cln2*^*+/+*^ (n = 3) and *Cln2*^*R207X/R207X*^ (n = 3) mice. Four technical replicates were performed using the three biological samples obtained from *Cln2*^*+/+*^ and *Cln2*^*R207X/R207X*^ mice. *Cln2*^*R207X/R207X*^ TPP1 activity was normalized to *Cln2*^*+/+*^ TPP1 activity. Columns and bars represent mean ± SEM. Statistical significance was determined using an unpaired t-test (** p < 0.01, ***p < 0.001, and **** p < 0.0001).

Transcripts containing PTCs that manage to escape NMD lead to the translation of truncated proteins; the functionality of these proteins is dependent on the location of the truncation. Therefore, in combination with NMD-mediated *Cln2* transcript reduction, the activity of TPP1, the enzyme encoded by *Cln2*, should be significantly reduced in *Cln2*^*R207X/R207X*^ mice. Using a previously established TPP1 fluorogenic enzyme activity assay, TPP1 activity was evaluated in *Cln2*^*R207X/R207X*^ mice (Fig 4). TPP1 activity was significantly reduced in five different tissues when compared to *Cln2*^*+/+*^ mice; the overall reduction consisted of at least 90%. To ensure that lysosomal dysfunction was solely due to reduced TPP1 activity, similar methods (fluorogenic enzyme activity assays) were used to assess the activity of palmitoyl-protein thioesterase 1 (PPT1), the lysosomal enzyme associated with cINCL, and sphingomyelinase, the lysosomal enzyme associated with Niemann-Pick disease. Overall, *Cln2*^*R207X/R207X*^ mice were found to have similar PPT1 and sphingomyelinase activity when compared to *Cln2*^*+/+*^ mice (S1 Fig). The only difference was found in the liver: a significant decrease in PPT1 activity in *Cln2*^*R207X/R207X*^ mice.

### Behavioral assessment of *Cln2*^*R207X/R207X*^ mice

**Fig 5:**
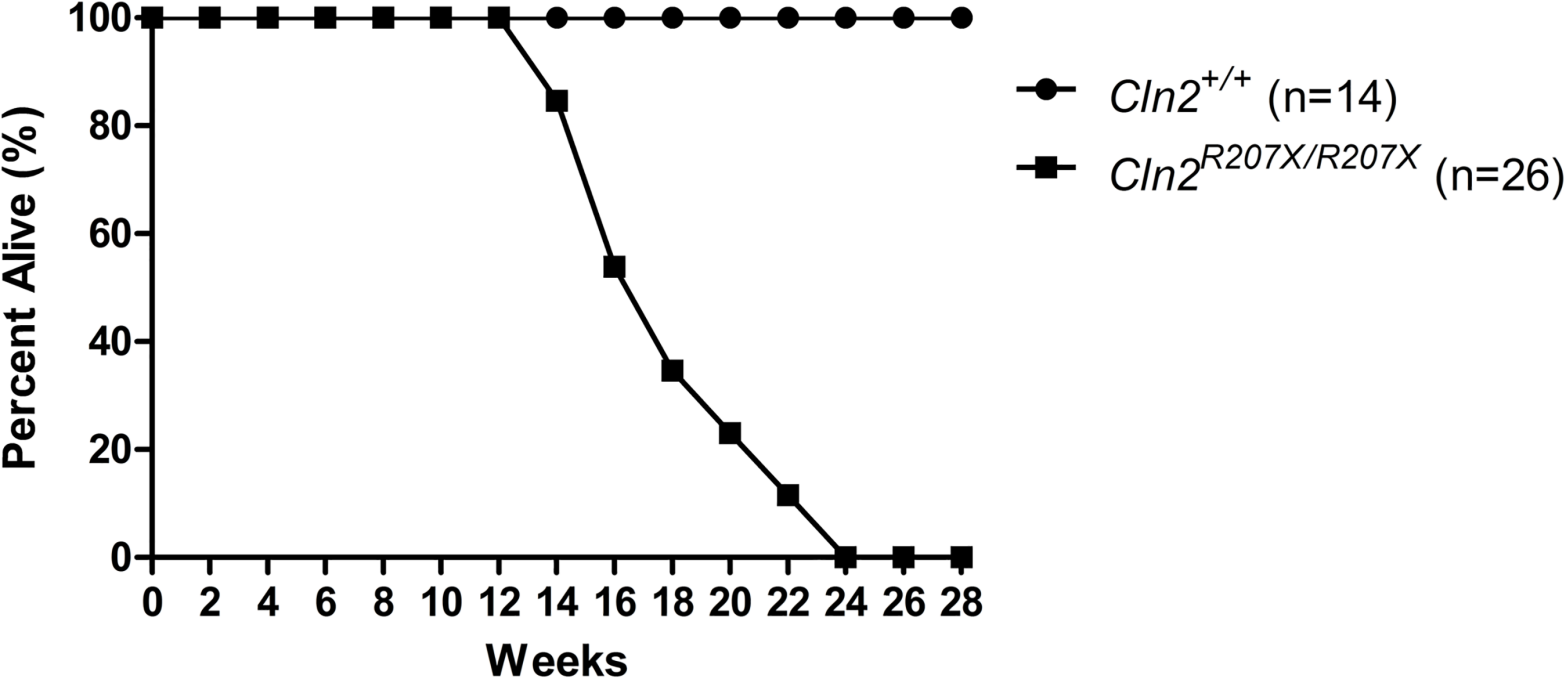
Significantly reduced lifespan in *Cln2*^*R207X/R207X*^ mice. *Cln2*^*+/+*^ (n = 14) and *Cln2*^*R207X/R207X*^ (n = 26) mice were plotted on a survival curve and tracked overtime. *Cln2*^*R207X/R207X*^ mice died between 3 and 6 months of age.

**Fig 6:**
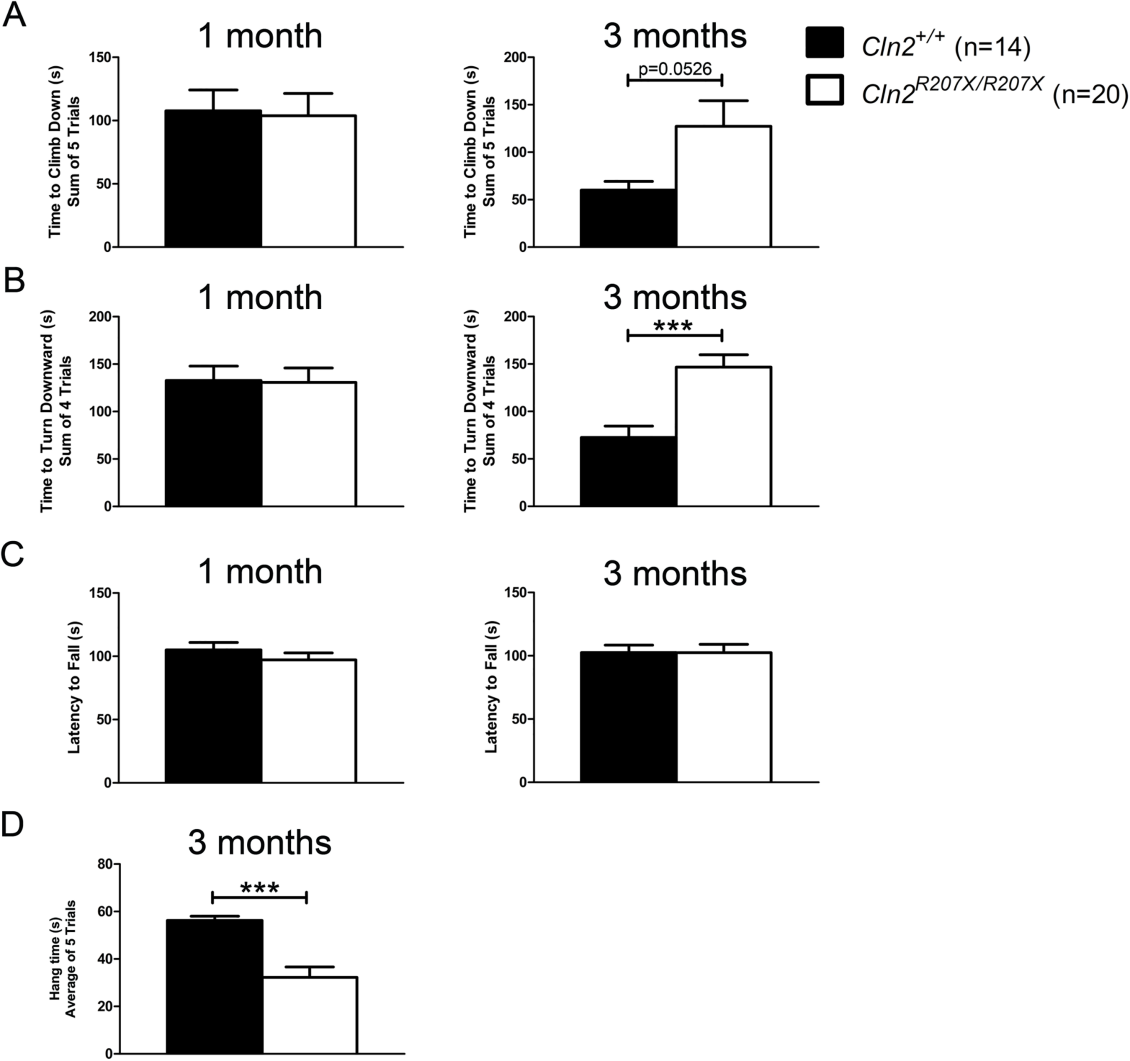
Altered motor skills in 3-month-old *Cln2*^*R207X/R207X*^ mice. A panel of behavioral tests including modified vertical pole (A and B), rotarod (C), and modified hanging wire (D) were used to assess motor skills in 1 and 3-month-old *Cln2*^*+/+*^ (n = 14) and *Cln2*^*R207X/R207X*^ (n = 20) mice. The modified hanging wire was only evaluated at 3 months of age. (A) Climb down time as assessed by the vertical pole showed no differences at 1 month of age between *Cln2*^*+/+*^ and *Cln2*^*R207X/R207X*^ but at 3 months of age *Cln2*^*R207X/R207X*^ mice trended towards an increased time. (B) Time to turn downward, also assessed by the vertical pole, showed no differences at 1 month of age between *Cln2*^*+/+*^ and *Cln2*^*R207X/R207X*^. By 3 months of age however, *Cln2*^*R207X/R207X*^ mice demonstrated significant difficulties turning around. (C) *Cln2*^*+/+*^ and *Cln2*^*R207X/R207X*^ mice exhibited no differences in their latency to fall from an accelerating rotarod at both 1 and 3 months of age. (D) 3-month-old *Cln2*^*R207X/R207X*^ mice have significant difficulties hanging from a wire rack. Columns and bars represent mean ± SEM. Statistical significance was determined using an unpaired t-test (***p < 0.001).

*Cln2*^*+/+*^ and *Cln2*^*R207X/R207X*^ male mice were compared in a battery of behavioral tests at 1 and 3 months of age. Older mice were not tested because premature death in *Cln2*^*R207X/R207X*^ mice started to appear after 3 months of age and progressively worsened; afflicting most mice by at least 6 months of age (Fig 5). To test for motor deficits, a modified vertical pole test was conducted as previously described [32]. First, the ability of a mouse to descend the pole was evaluated in 5 consecutive trials, and then the ability of the same mouse to turn around on the pole was tested in 4 consecutive trials. At 1 month of age, both *Cln2*^*+/+*^ and *Cln2*^*R207X/R207X*^ mice descended the vertical pole in similar time (Fig 6A). At 3 months, *Cln2*^*R207X/R207X*^ mice strongly trended towards taking a longer time to descend but the difference between *Cln2*^*+/+*^ and *Cln2*^*R207X/R207X*^ mice was shy of statistical significance (Fig 6A). In terms of their ability to completely turn around on the vertical pole, while no differences were detected at 1 month, *Cln2*^*R207X/R207X*^ mice at 3 months displayed significant difficulties (Fig 6B). Motor coordination and balance was also tested in an accelerating rotarod test (0-48rpm in 240 sec). At 1 and 3 months, the rotarod performance of *Cln2*^*+/+*^ and *Cln2*^*R207X/R207X*^ mice was similar (Fig 6C). Given that 3-month-old *Cln2*^*R207X/R207X*^ mice started to display some motor deficits, overall strength was evaluated using the hanging wire test. At 3 months of age, *Cln2*^*R207X/R207X*^ mice showed significant impediments in their ability to hang from a wire rack (Fig 6D). To examine if anxiety is a contributing factor to the aforementioned behaviors, the anxiety level of 3-month-old *Cln2*^*+/+*^ and *Cln2*^*R207X/R207X*^ mice were evaluated using the light/dark box test. No difference in anxiety between Cln2^*+/+*^ and *Cln2*^*R207X/R207X*^ mice was found (S2 Fig).

**Fig 7:**
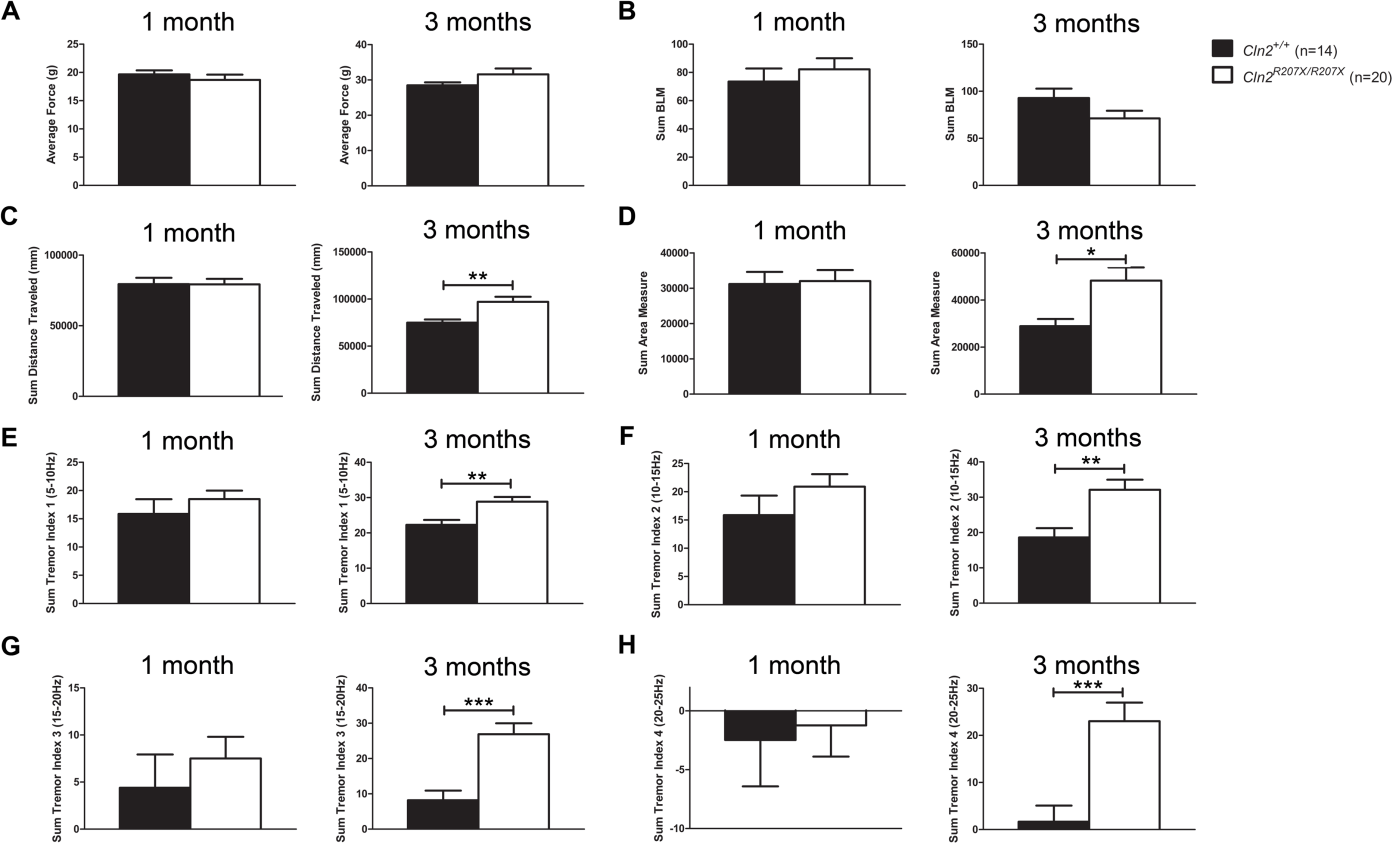
*Cln2*^*R207X/R207X*^ mice display characteristics of hyperactivity and develop tremors by 3 months of age. Various behavioral characteristics of 1 and 3-month-old *Cln2*^*+/+*^ (n = 14) and *Cln2*^*R207X/R207X*^ (n = 20) mice were evaluated using a force-plate actimeter. (A) Average force increased from 1 to 3 months of age but did not differ between *Cln2*^*+/+*^ and *Cln2*^*R207X/R207X*^ at either time points. (B) *Cln2*^*+/+*^ and *Cln2*^*R207X/R207X*^ mice had similar bouts of low mobility at both 1 and 3 months. (C,D) By 3 months of age, *Cln2*^*R207X/R207X*^ mice display signs of hyperactivity such as significantly increased distance traveled and area coverage. (E-H) Software analysis of the force-plate actimeter data permits the evaluation of tremors by calculating a tremor index score. Tremor index scores are separated according to tremor frequencies such as, 5–10 Hz (E), 10–15 Hz (F), 15–20 Hz (G) and 20–25 Hz (H). As *Cln2*^*R207X/R207X*^ mice aged from 1 to 3 months, tremor index scores increased significantly. Columns and bars represent mean ± SEM. Statistical significance was determined using an unpaired t-test (*p < 0.05, **p < 0.01, and ***p < 0.001).

To test multiple behavioral parameters in freely-moving *Cln2*^*+/+*^ and *Cln2*^*R207X/R207X*^ mice a force-plate actimeter was used (BASi, West Lafayette, IN; Ossowska et al., 2014, Neuropharmacology 83: 28–35). This device tracks the movement of mice by tracing the changes in the force applied to the plate. Several parameters including distance traveled, area covered, number of left and right turns, focused stereotypes, low mobility bouts, and tremor index are measured. The average force, closely related to the weight of the mice, did not differ between *Cln2*^*+/+*^ and *Cln2*^*R207X/R207X*^ mice at 1 and 3 months (Fig 7A). *Cln2*^*+/+*^ and *Cln2*^*R207X/R207X*^ mice also had similar bouts of low mobility at both 1 and 3 months (Fig 7B). Tracings of the mice indicated that at 3 months, *Cln2*^*R207X/R207X*^ mice traveled a greater distance and covered a larger surface area (Fig 7C and 7D). These potential indicators of hyperactivity correlated with observations made during the handling of the mice. Through continuous tracking of the applied force, tremors or seizures can be detected and measured by calculating a tremor index score. Tremor indices for this study covered tremor frequencies ranging from 5–10 Hz, 10–15 Hz, 15–20 Hz, and 20–25 Hz. At 1 month of age, *Cln2*^*+/+*^ and *Cln2*^*R207X/R207X*^ mice had similar tremor index scores for all frequency ranges (Fig 7E–7H). At 3 months of age, however, *Cln2*^*R207X/R207X*^ mice showed a marked increase in all four tremor indices (Fig 7E–7H). Observationally, the tremor became more severe and debilitating after 3 months of age.

### Histological evaluation of *Cln2*^*R207X/R207X*^ mice

**Fig 8:**
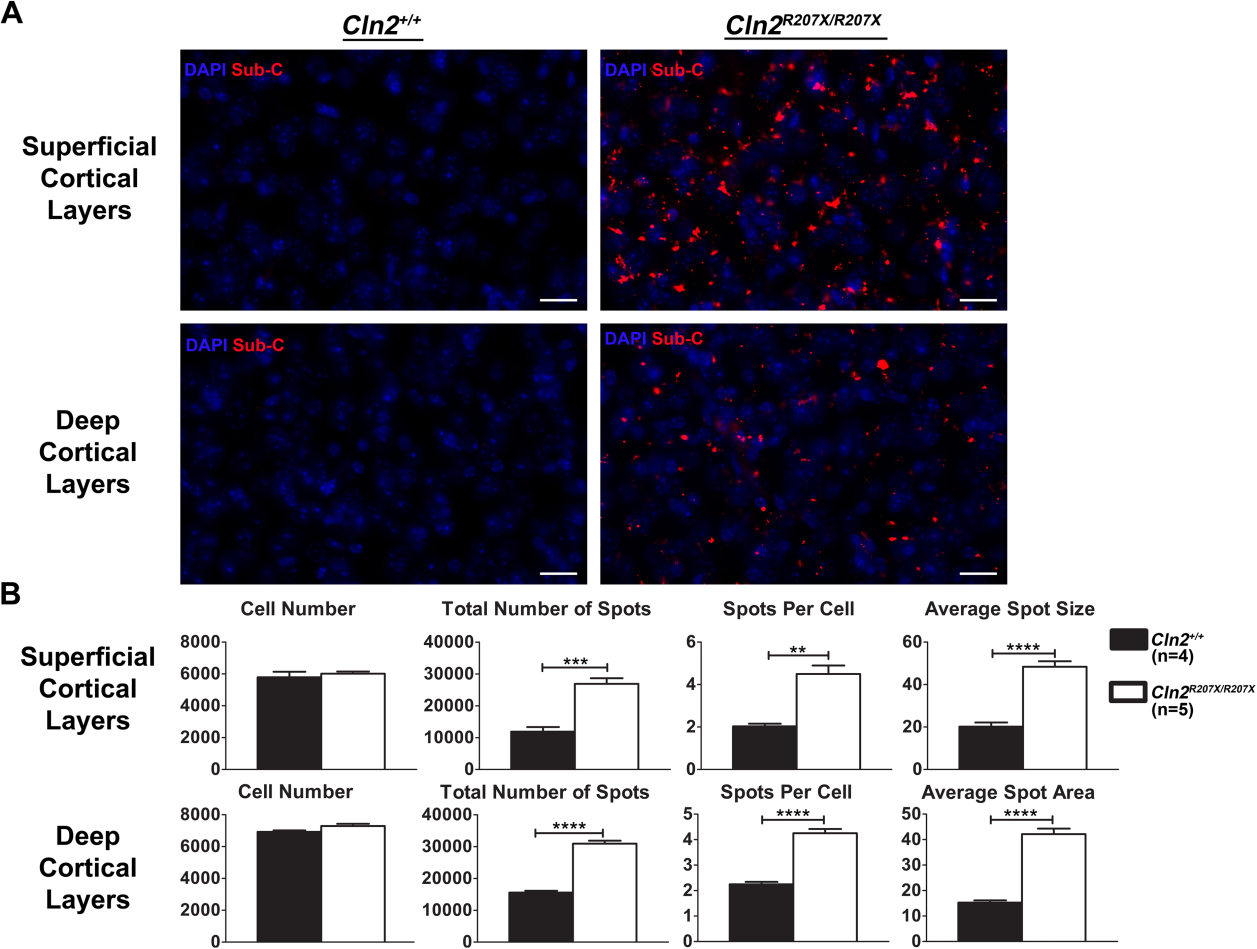
Lysosomal accumulation of mitochondrial ATP synthase subunit c in *Cln2*^*R207X/R207X*^ mice. Subunit c accumulation was detected by immunofluorescent staining. (A) Images of superficial and deep cortical layers demonstrate diffuse and pronounced accumulation of mitochondrial ATP synthase subunit c in 3-month-old *Cln2*^*R207X/R207X*^ mice. (B) Images from 3-month-old *Cln2*^*+/+*^ (n = 4) and *Cln2*^*R207X/R207X*^ (n = 5) mice were blindly collected and analyzed for differences in cell number, total number of immunoreactive puncta, number of puncta per cell, and average punctum area. *Cln2*^*R207X/R207X*^ mice have significantly increased total number of puncta, puncta per cell, and punctum size. Columns and bars represent mean ± SEM. Statistical significance was determined using an unpaired t-test (**p < 0.01, ***p < 0.001, and **** p < 0.0001).

Based on the age of onset for behavioral anomalies in the *Cln2*^*R207X/R207X*^ mice, 3-month-old *Cln2*^*R207X/R207X*^ cerebrums and cerebellums were evaluated for NCL associated neuropathological hallmarks. Generally, these include lysosomal storage material accumulation, astrocytosis, and activated microglia [11, 17, 33–39]. Due to the natural fluorescence of NCL-associated lysosomal storage material, intracellular autofluorescence is usually measured. Three-month-old *Cln2*^*R207X/R207X*^ cerebrum and cerebellum did not demonstrate autofluorescent storage material (data not shown). Nevertheless, a principal component of cLINCL storage material is mitochondrial ATP synthase subunit c, a substrate of TPP1 [40–43]. Using immunohistochemistry, *Cln2*^*+/+*^ and *Cln2*^*R207X/R207X*^ brain sections were evaluated for mitochondrial ATP synthase subunit c accumulation. The cerebral cortex of *Cln2*^*R207X/R207X*^ mice had a pronounced accumulation of mitochondrial ATP synthase subunit c throughout superficial and deep cortical layers; whereas, *Cln2*^*+/+*^ brain sections had little immunoreactivity (Fig 8A). Quantitative analysis of the immunoreactive puncta throughout the *Cln2*^*R207X/R207X*^ cerebral cortex in comparison to the *Cln2*^*+/+*^ cortex revealed an increased frequency per tissue (superficial layers: 2.26 fold increase, p = 0.0004; deep layers: 1.98 fold increase, p < 0.0001), increased frequency per cell (superficial layers: 2.21 fold increase, p = 0.0011; deep layers: 1.89 fold increase, p < 0.0001), and a greater size (superficial layers: 2.4 fold increase, p < 0.0001; deep layers: 2.67 fold increase, p < 0.0001) (Fig 8B). Likewise, the cerebellum of *Cln2*^*R207X/R207X*^ mice had profound mitochondrial ATP synthase subunit c accumulation throughout cerebellar regions, whereas the cerebellum of *Cln2*^*+/+*^ mice had little to none (S3 Fig). To confirm the subcellular location of the ATP synthase subunit c puncta, cerebral cortical sections from *Cln2*^*R207X/R207X*^ mice were immunostained for LAMP-1, a lysosomal membrane protein, and for the mitochondrial ATP synthase subunit c. A significant portion of mitochondrial ATP synthase subunit c co-localized with LAMP-1 (S4 Fig).

**Fig 9:**
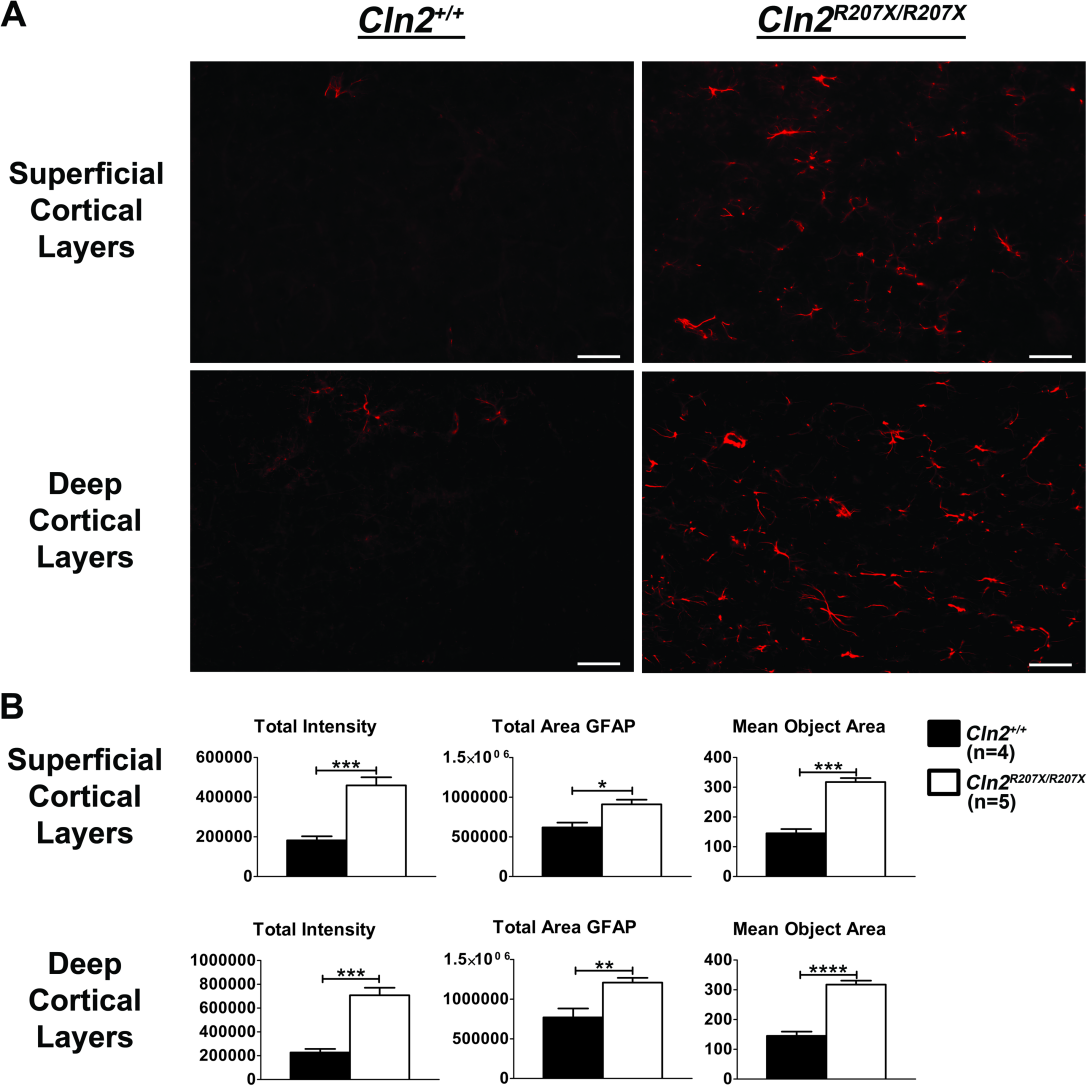
Diffuse cortical astrocytosis in *Cln2*^*R207X/R207X*^ mice. (A) Images of superficial and deep cortical layers from 3-month-old *Cln2*^*+/+*^ and *Cln2*^*R207X/R207X*^ mice demonstrate increased GFAP immunostaining and enlarged astrocytes. (B) Images from 3-month-old *Cln2*^*+/+*^ (n = 4) and *Cln2*^*R207X/R207X*^ (n = 5) mice were blindly collected and analyzed for GFAP immunostaining intensity, GFAP staining area, and astrocyte area. *Cln2*^*R207X/R207X*^ mice have significantly increased GFAP immunostaining and astrocyte size relative to *Cln2*^*+/+*^. Columns and bars represent mean ± SEM. Statistical significance was determined using an unpaired t-test (*p < 0.05, **p < 0.01, ***p < 0.001, and **** p < 0.0001).

Astrocytosis is a common pathological finding in neurodegenerative diseases [44–47]. Most commonly, the immunostaining intensity of glial fibrillary acidic protein (GFAP) and astrocyte morphology are used to evaluate astrocytosis; even within the NCLs [11, 17, 33–36, 38, 48–51]. Therefore, 3-month-old *Cln2*^*+/+*^ and *Cln2*^*R207X/R207X*^ cerebral sections were immunostained for GFAP. Superficial and deep cortical layers in sections from *Cln2*^*R207X/R207X*^ mice demonstrated significantly increased GFAP immunostaining as well as hypertrophy of astrocytes when compared to sections from *Cln2*^*+/+*^ mice (Fig 9A). Superficial cortical layers of *Cln2*^*R207X/R207X*^ mice were found to have a 2.52 fold increase in GFAP intensity (p = 0.0008) and a 2.04 fold increase in mean astrocyte area (p = 0.0002), whereas deep cortical layers of *Cln2*^*R207X/R207X*^ mice showed a 3.12 fold increase in GFAP intensity (p = 0.0004) and a 2.19 fold increase in mean astrocyte area (p < 0.0001) (Fig 9B). An additional pathological feature of neurodegenerative diseases is activated microglia. Microglial activation in the brain can be evaluated using immunostaining for the microglial markers, Iba1, F4/80, and CD68 [52–56]. The morphology of microglial cells dramatically changes during activation: the resting cells lose their processes and become more rounded [52, 53, 57]. Cerebral sections from 3-month-old *Cln2*^*+/+*^ and *Cln2*^*R207X/R207X*^ mice were immunostained for Iba1, and microglial activation was evaluated based on staining intensity and cell morphology. No detectable differences were identified in total Iba1 immunostaining intensity, average microglial Iba1 intensity, and average microglia size (S5 Fig). Although microglial activation in *Cln2*^*R207X/R207X*^ mice was not present at 3 months, microglial activation during later periods of disease progression cannot be ruled out. However, for the purposes of this project we were interested in brain pathology prior to the sudden decline in the survival curve.

Collectively, cerebral and cerebellar lysosomal accumulation of mitochondrial ATP synthase subunit c and cerebral astrocytosis indicate disease pathology at the onset of both behavioral phenotypes and decline in survival. However, these pathological findings do not identify a clear cause of premature death. To help identify potential causes of death and peripheral pathology, tissues (i.e. spleen, lung, heart, eye, and brain) from 4-month-old *Cln2*^*+/+*^ and *Cln2*^*R207X/R207X*^ mice were sectioned, hematoxylin/eosin stained, and sent to a veterinary pathologist for examination. The veterinary pathologist was blinded from all identifying information. According to the pathology report, the brain was the only tissue with identifiable abnormalities which consisted of hypereosinophilic granules and sporadic neurodegeneration (S6 Fig). Consequently, no definitive causes of death were identified.

## Discussion

Current preclinical therapeutic studies for CLN2 disease utilize the *Cln2*^*-/-*^ mouse model which carries an uncommon missense mutation [37]. *Cln2*^*-/-*^ mice are not suitable to test therapies such as read-through compounds, nonsense mediated decay inhibitors, and clinically relevant mutation targeting antisense oligonucleotides. Therefore, the primary purpose of this study was to generate an alternative model of CLN2 disease that allows for the evaluation of all therapies including those previously excluded.

The new model was chosen to have the most common nonsense mutation that occurs in CLN2 disease patients, p.R207X. To be considered a model of CLN2 disease, the *Cln2*^*R207X/R207X*^ mouse was rigorously characterized to confirm the presence of CLN2 disease-associated anomalies. Molecular and biochemical characterization confirmed that the presence of the p.R207X mutation targeted *Cln2* transcripts for the governing nonsense mediated decay pathway; this reduction in combination with TPP1 truncation resulted in a significant decrease in ubiquitous TPP1 activity. These alterations in TPP1 activity are similar to what is seen in CLN2 disease patients [25, 58, 59]. Loss of TPP1 activity results in lysosomal dysfunction. Thus, the lysosomes in CLN2 disease patients and models accumulate autofluorescent storage material which consists of various components including mitochondrial ATPase synthase subunit c [40, 60–62]. Histological assessment of *Cln2*^*R207X/R207X*^ cerebral and cerebellar sections did not show autofluorescent storage material at the same time point as symptomatic onset (3 months). Nevertheless, similar sections strongly demonstrated the accumulation of mitochondrial ATPase synthase subunit c. Co-localization of mitochondrial ATPase synthase subunit c and LAMP-1 indicates lysosomal accumulation of non-autofluorescent material prior to the accumulation of autofluorescent components. Secondary pathological findings such as astrocytosis in 3-month-old *Cln2*^*R207X/R207X*^ cerebral sections confirm previous findings of NCL. Overall, both biochemical and histological assessments of *Cln2*^*R207X/R207X*^ mice demonstrate similar findings to the *Cln2*^*-/-*^ mouse model and CLN2 disease patients.

Severe deficiency in TPP1 activity and neuropathological abnormalities lead CLN2 disease patients to develop incapacitating neurological deficits and ultimately death [9, 16]. Behavioral characterization of *Cln2*^*R207X/R207X*^ mice identified motor and strength deficits starting at 3 months of age. Unfortunately most *Cln2*^*R207X/R207X*^ mice did not survive to be tested for motor coordination at either 4 or 5 months, those that could be tested failed immediately. In addition to motor and strength deficits, *Cln2*^*R207X/R207X*^ mice at 3 months of age developed a tremor that progressively worsened and became significantly debilitating. This behavioral anomaly is being characterized as a tremor based on presentation; although it cannot be ruled out as seizure activity without further specialized testing. Overall, these behavioral abnormalities contribute to a significant shortening of the *Cln2*^*R207X/R207X*^ mouse lifespan. We tried to test visual acuity (e.g. visual cliff and mouse optometry system) in 1 and 3 month old wildtype and mutant mice. However, the hyperactivity of the mutant mice prohibited adequate assessment of visual acuity. *Cln2*^*R207X/R207X*^ mice will be given to an expert in NCL vision loss and retinal pathology to investigate visual changes in these new *Cln2* mutant mice.

Collectively, the biochemical, histological, and behavioral characterization of *Cln2*^*R207X/R207X*^ mice clearly demonstrates the successful generation of a new CLN2 disease model. Unlike previous mouse models, this model carries a common disease causing CLN2 mutation. In addition, this model allows for the assessment of all therapies. Preclinical studies investigating the use of read through compounds and nonsense mediated decay inhibitors have shown positive outcomes in other disease models including lysosomal storage disorders [23, 25, 63–69]. Prior to the development of our *Cln2*^*R207X/R207X*^ mouse model, preclinical studies into these therapies could not be evaluated for CLN2 disease. The generation of this new CLN2 disease model advances preclinical studies into CLN2 disease therapies.

## Supporting information

S1 Fig Unaltered sphingomyelinase activity and selectively altered PPT1 activity in various *Cln2*^*R207X/R207X*^ mouse tissues. Fluorogenic enzyme activity assays for sphingomyelinase (A) and PPT1 (B) were used to measure endogenous activity in five different tissues from 1-month-old *Cln2*^*+/+*^ (n = 3) and *Cln2*^*R207X/R207X*^ (n = 3) mice. In the sphingomyelinase activity assay, three technical replicates were performed using the three biological samples obtained from *Cln2*^*+/+*^ and *Cln2*^*R207X/R207X*^ mice; whereas, the PPT1 activity assays used four technical replicates from the three biological samples. *Cln2*^*R207X/R207X*^ sphingomyelinase and PPT1 activity was normalized to *Cln2*^*+/+*^ activity levels. Columns and bars represent mean ± SEM. Statistical significance was determined using an unpaired t-test (*p < 0.05).
(TIF)

S2 Fig Similar levels of anxiety in *Cln2*^*+/+*^ and *Cln2*^*R207X/R207X*^ mice. The light/dark box test was used to assess for anxiety in 3-month-old *Cln2*^*+/+*^ (n = 14) and *Cln2*^*R207X/R207X*^ (n = 20) mice. Both cohorts spent similar amounts of time in the dark. Columns and bars represent mean ± SEM. Statistical significance was determined using an unpaired t-test.
(TIF)

S3 Fig Cerebellar layers of *Cln2*^*R207X/R207X*^ mice display enhanced mitochondrial ATP synthase subunit c accumulation. Images of the molecular (ML) and granular (GL) cerebellar layers demonstrate accumulation of mitochondrial ATP synthase subunit c in *Cln2*^*R207X/R207X*^ mice when compared to *Cln2*^*+/+*^ controls. Pronounced accumulation is present in the Purkinje cell layer (at the border of ML and GL) in *Cln2*^*R207X/R207X*^ mice.
(TIF)

S4 Fig A significant portion of mitochondrial ATP synthase subunit c accumulation is localized to the lysosome. *Cln2*^*R207X/R207X*^ cerebral sections immunostained with anti-subunit c (red) and anti-LAMP-1 (green) reveal co-localization.
(TIF)

S5 Fig No signs of activated microglia at 3 months in *Cln2*^*R207X/R207X*^ mice. (A) Superficial and deep cortical layers from 3-month-old *Cln2*^*+/+*^ and *Cln2*^*R207X/R207X*^ mice show minimal differences in immunostaining for the microglial marker, Iba1. (B) Images from 3-month-old *Cln2*^*+/+*^ (n = 4) and *Cln2*^*R207X/R207X*^ (n = 5) mice were blindly collected and analyzed for Iba1 immunostaining total intensity, average intensity per Iba1 positive microglia, and average microglia size. Columns and bars represent mean ± SEM. Statistical significance was assessed using an unpaired t-test.
(TIF)

S6 Fig Hypereosinophilic inclusions and neurodegeneration in 4-month-old *Cln2*^*R207X/R207X*^ mice. Tissues from *Cln2*^*+/+*^ (n = 3) and *Cln2*^*R207X/R207X*^ (n = 3) mice were sectioned, hematoxylin/eosin stained, and evaluated by a veterinary pathologist under blinded conditions. The pathology report solely identified cerebral cellular hypereosinophilic inclusions (arrows) and signs of neurodegeneration (arrow heads) in *Cln2*^*R207X/R207X*^ mice (A-C) when compared to *Cln2*^*+/+*^ controls (D).
(TIF)

S7 Fig Guidelines checklist. (PDF)

## Abbreviations

LSD: lysosomal storage disorder
cINCL: classic infantile neuronal ceroid lipofuscinosis
cLINCL: classic late infantile neuronal ceroid lipofuscinosis
cJNCL: classic juvenile neuronal ceroid lipofuscinosis
TPP1: tripeptidyl-peptidase 1
PPT1: palmitoyl protein thioesterase 1
Gapdh: glyceraldehyde-3-phosphate dehydrogenase
GFAP: glial fibrillary acidic protein
Iba1: ionized calcium binding adapter molecule 1
DAPI: 4’,6-Diamidino-2-Phenylindole
