# Supplementary figures and images for: A tailored mouse model of CLN2 disease: A nonsense mutant for testing personalized therapies
Source: PLoS One. 2017 May 2;12(5):e0176526. doi: 10.1371/journal.pone.0176526 (PMC5413059; doi:10.1371/journal.pone.0176526)

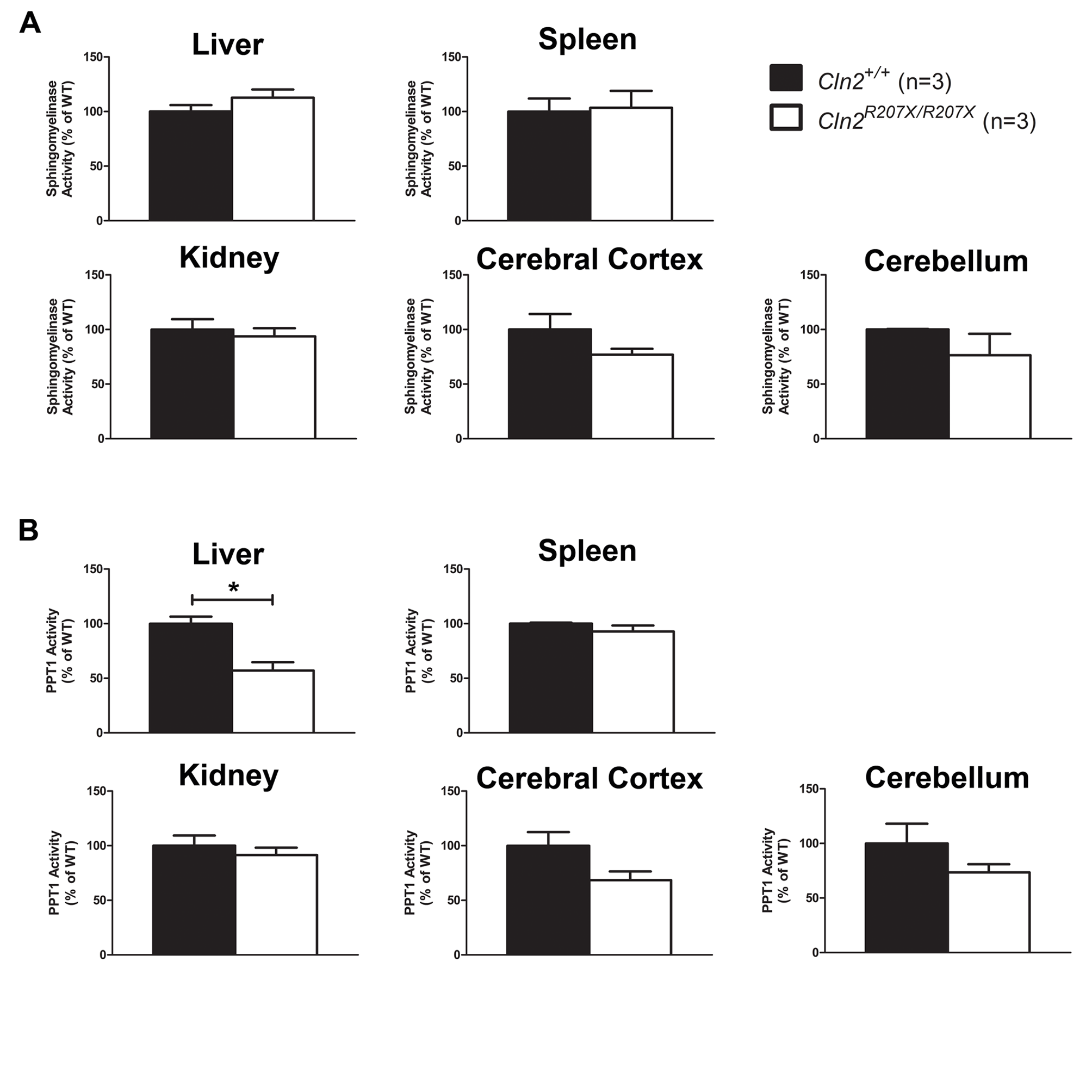

Supplement: S1 Fig — Fluorogenic enzyme activity assays for sphingomyelinase (A) and PPT1 (B) were used to measure endogenous activity in five different tissues from 1-month-old Cln2+/+ (n = 3) and Cln2R207X/R207X (n = 3) mice. In the sphingomyelinase activity assay, three technical replicates were performed using the three biological samples obtained from Cln2+/+ and Cln2R207X/R207X mice; whereas, the PPT1 activity assays used four technical replicates from the three biological samples. Cln2R207X/R207X sphingomyelinase and PPT1 activity was normalized to Cln2+/+ activity levels. Columns and bars represent mean ± SEM. Statistical significance was determined using an unpaired t-test (*p < 0.05). (TIF) [file pone.0176526.s001.tif]

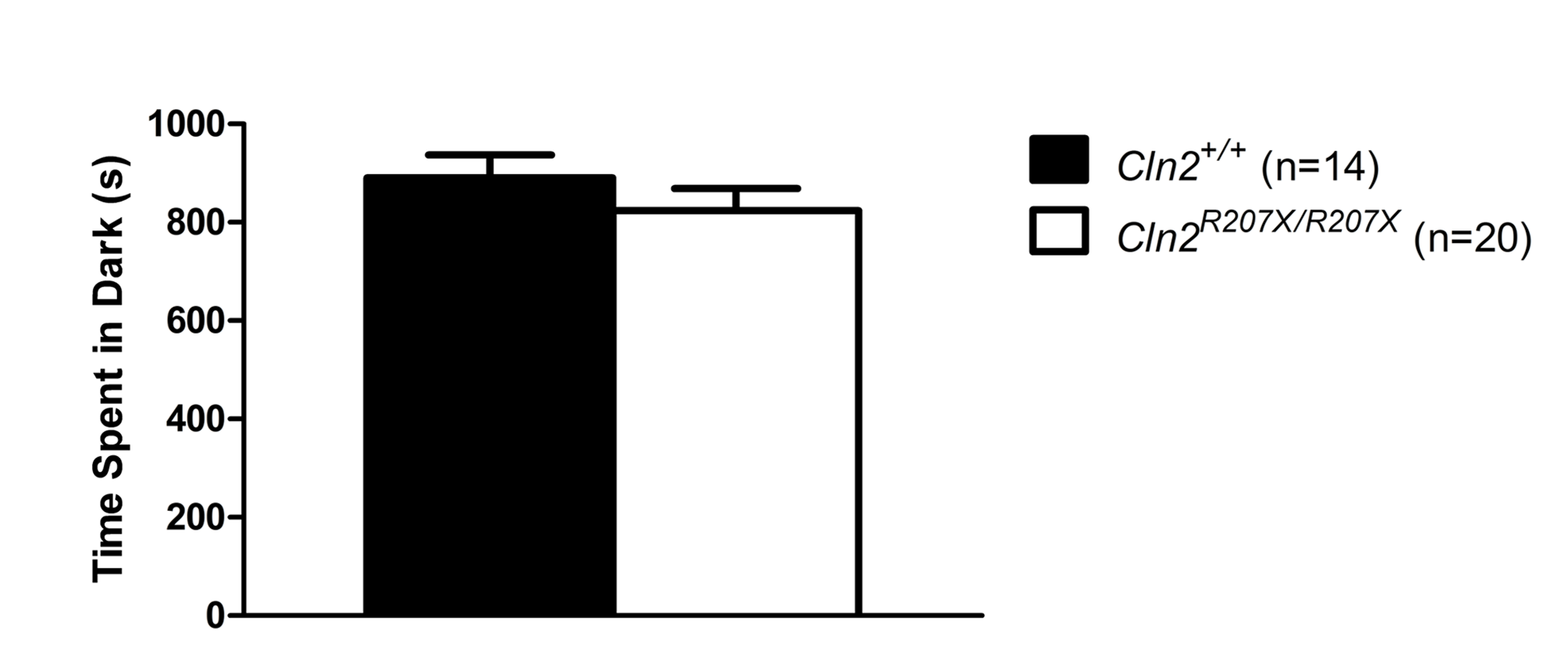

Supplement: S2 Fig — The light/dark box test was used to assess for anxiety in 3-month-old Cln2+/+ (n = 14) and Cln2R207X/R207X (n = 20) mice. Both cohorts spent similar amounts of time in the dark. Columns and bars represent mean ± SEM. Statistical significance was determined using an unpaired t-test. (TIF) [file pone.0176526.s002.tif]

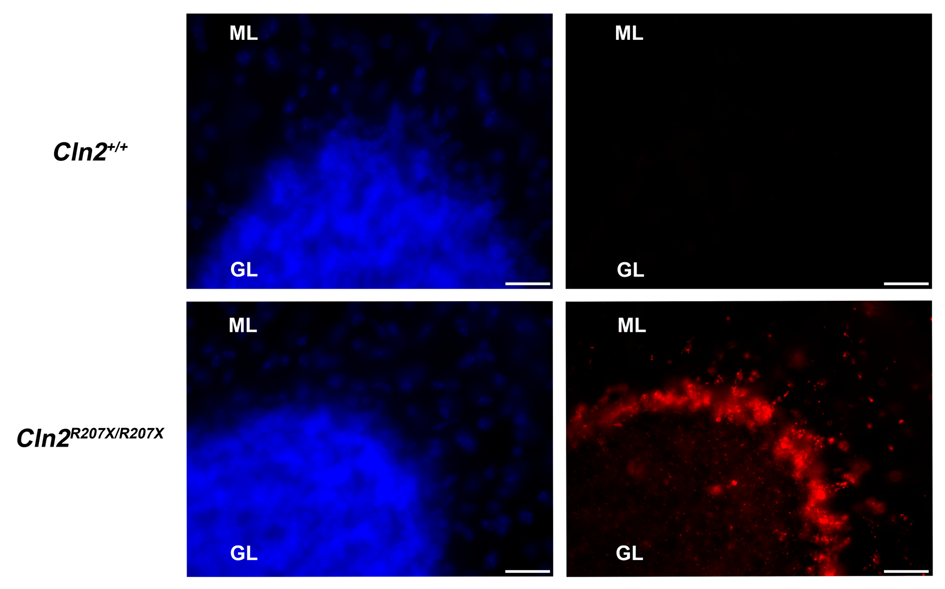

Supplement: S3 Fig — Images of the molecular (ML) and granular (GL) cerebellar layers demonstrate accumulation of mitochondrial ATP synthase subunit c in Cln2R207X/R207X mice when compared to Cln2+/+ controls. Pronounced accumulation is present in the Purkinje cell layer (at the border of ML and GL) in Cln2R207X/R207X mice. (TIF) [file pone.0176526.s003.tif]

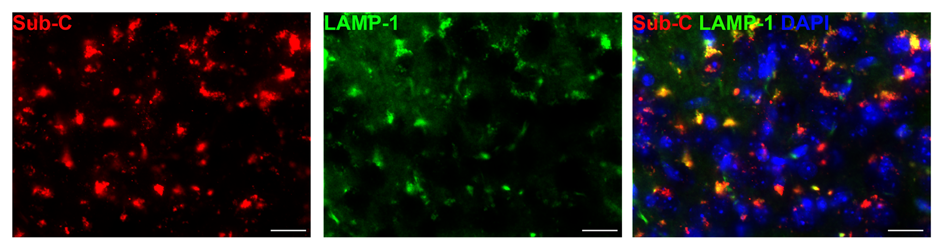

Supplement: S4 Fig — Cln2R207X/R207X cerebral sections immunostained with anti-subunit c (red) and anti-LAMP-1 (green) reveal co-localization. (TIF) [file pone.0176526.s004.tif]

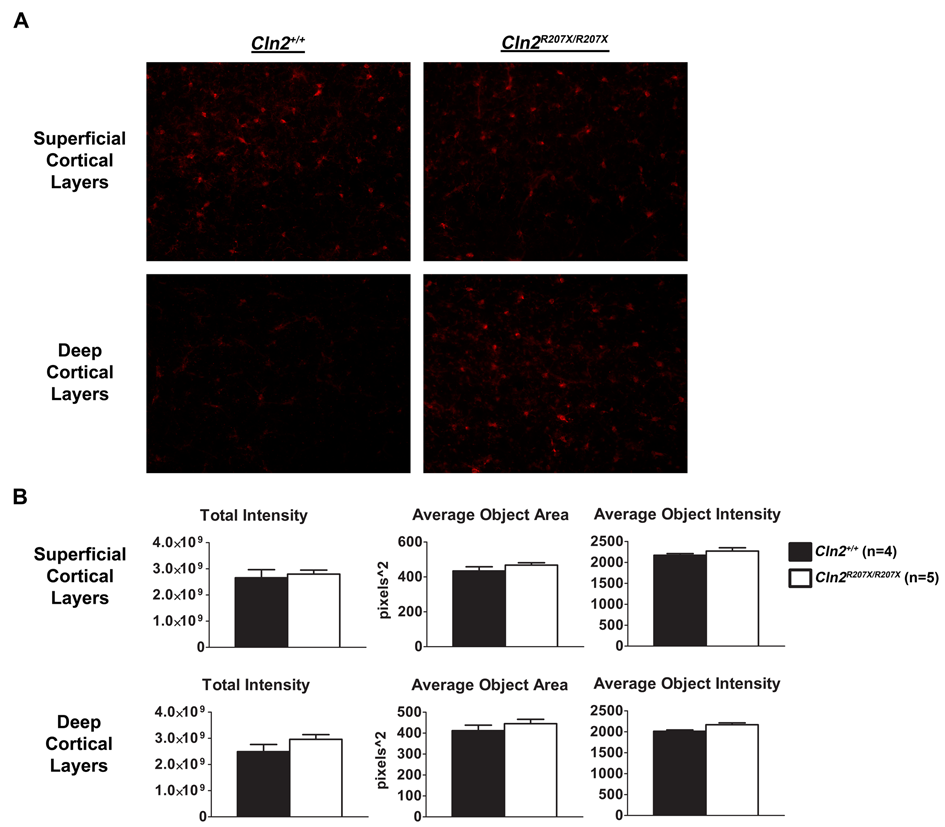

Supplement: S5 Fig — (A) Superficial and deep cortical layers from 3-month-old Cln2+/+ and Cln2R207X/R207X mice show minimal differences in immunostaining for the microglial marker, Iba1. (B) Images from 3-month-old Cln2+/+ (n = 4) and Cln2R207X/R207X (n = 5) mice were blindly collected and analyzed for Iba1 immunostaining total intensity, average intensity per Iba1 positive microglia, and average microglia size. Columns and bars represent mean ± SEM. Statistical significance was assessed using an unpaired t-test. (TIF) [file pone.0176526.s005.tif]

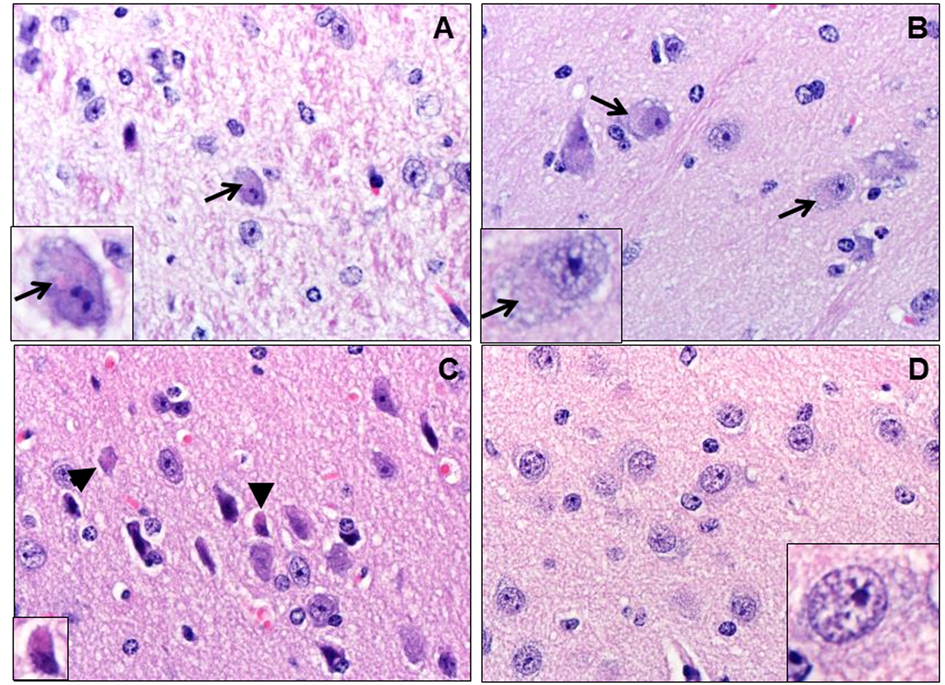

Supplement: S6 Fig — Tissues from Cln2+/+ (n = 3) and Cln2R207X/R207X (n = 3) mice were sectioned, hematoxylin/eosin stained, and evaluated by a veterinary pathologist under blinded conditions. The pathology report solely identified cerebral cellular hypereosinophilic inclusions (arrows) and signs of neurodegeneration (arrow heads) in Cln2R207X/R207X mice (A-C) when compared to Cln2+/+ controls (D). (TIF) [file pone.0176526.s006.tif]
